# Teprotumumab: a disease modifying treatment for graves’ orbitopathy

**DOI:** 10.1186/s13044-020-00086-7

**Published:** 2020-07-04

**Authors:** Michelle Ting, Daniel G. Ezra

**Affiliations:** 1 Adnexal Department, Moorfields Eye Hospital NHS Foundation Trust, 162 City Road, London, EC1V 2PD UK; 2 UCL Institute of Ophthalmology and Moorfields Eye Hospital NIHR Biomedical Research Centre for ophthalmology, London, UK

**Keywords:** Teprotumumab, Graves’ orbitopathy, Thyroid eye disease, Proptosis, Monoclonal antibodies, Insulin-like growth Factor-1 receptor, Diplopia

## Abstract

**Background:**

On 21st January 2020, the FDA approved Tepezza (teprotumumab-trbw) for the treatment of active Graves’ orbitopathy (GO) in adults. This approval was based on positive results from two multinational randomised double-blind placebo-controlled clinical trials.

**Discussion:**

This article discusses the outcomes of those trials and the potential role of teprotumumab in altering current treatment paradigms in Graves’ orbitopathy. Future challenges are explored, including the need to confirm its disease-modifying effect, to establish its optimal position in the treatment pathway, and to define the appropriate subset of patients who would benefit from its use.

**Conclusions:**

The results from these two large clinical trials have shown teprotumumab to have remarkable effects on multiple clinical outcomes in GO, particularly in its ability to reverse proptosis. It may herald a new era in the treatment of thyroid eye disease and could offer an alternative to surgery and its associated complications. Additional studies will continue to shape the treatment of GO and define the role of teprotumumab within the treatment paradigm.

## Introduction

On 21st January 2020, the FDA approved Tepezza (teprotumumab-trbw) for the treatment of active Graves’ orbitopathy (GO) in adults in the US [1]. This represents the first drug approval for the treatment of GO and is based on positive results from two multinational randomised double-blind placebo-controlled clinical trials [2, 3].

TED is an autoimmune inflammatory condition, affecting up to 50% of patients with Graves’ hyperthyroidism and occasionally affecting patients with other forms of autoimmune thyroiditis [4]. The majority of patients experience a mild disease course requiring conservative treatment only, but up to 33% develop moderate-to-severe disease [5], characterized by diplopia and marked proptosis, which are associated with reduced quality of life [6]. The worst cases develop sight-threatening complications including compressive optic neuropathy or exposure keratopathy [7]. Its clinical course typically follows a pattern originally described by Rundle and Wilson [8], with an initial active phase, characterized by evolving symptoms and signs of inflammation of the periocular soft tissues. Patients in the active phase can exhibit orbital pain, lid swelling and erythema, conjunctival redness and chemosis, and enlargement of the extraocular muscles and the orbital fatty volume resulting in proptosis. A ‘Clinical Activity Score (CAS)’ can be created by tallying the patient’s inflammatory symptoms and signs; this acts an aid to monitoring the patient’s disease progression over time [9]. Following the inflammatory phase, patients enter the ‘burnt out’ inactive phase with subsequent tissue remodelling and fibrosis. Once in this phase, long term sequelae such as proptosis or diplopia can be addressed with multi-staged rehabilitative surgery, including orbital decompression, strabismus surgery and lid surgery [10].

Although the pathogenesis of GO is not completely understood, it is known that a central role is played by orbital fibroblasts expressing TSH receptors that become activated by TSH receptor autoantibodies. This results in the release of proinflammatory mediators, with changes to extracellular matrix components and enhanced adipogenesis, contributing to proptosis. I*n vitro*, a subpopulation of orbital fibroblasts has the potential to differentiate into mature adipocytes, and these could contribute to increased adipose tissue in vivo [11]. An important role is also played by the insulin-like growth factor-1 receptor (IGF-1R) which appears to modulate and enhance the pathogenic actions of TSH-receptor antibodies on the TSH receptor [12].

## Conventional treatments for GO

The management of moderate to severe GO is challenging, requiring a multidisciplinary team of both endocrinologists and ophthalmologists. Current treatment strategies focus on immune suppression in the active phase in patients with moderate-to-severe disease [13]. The mainstay of these is steroids, with intravenous pulsed glucocorticoids being preferred over oral administration due to a more favorable safety and efficacy profile [14]. Although trends and preferences for the use of steroids vary between regions, (European clinicians are more in favour of steroid use for active GO than their North American counterparts due to EUGOGO recommendations), trials show that steroid treatment can result in a clinically meaningful improvement in the Clinical Activity Score [12, 15]. However, the only published placebo-controlled steroid trial showed that intravenous methylprednisolone does not significantly improve measures of proptosis or diplopia [16]. Furthermore, high dose glucocorticoid therapy can have undesired adverse effects [14, 15]. Orbital radiotherapy is sometimes used in combination with steroids to reduce motility impairment but does not have any effect on proptosis, disease progression and quality of life [17, 18].

If there is an inadequate response to glucocorticoid therapy, several second-line therapies are available, including cyclosporine [19], methotrexate [20], azathioprine [21], somatostatin analogues [22], mycophenolate mofetil [23], tocilizumab [24] and rituximab [25]. Like steroids, these treatments do not significantly alter long term disease outcomes [14]. Whilst existing treatments may improve inflammatory activity, they do not reduce the need for subsequent rehabilitative surgery once patients reach the fibrotic stage of the disease.

## Teprotumumab: a new era?

Developments in the understanding of the molecular basis of GO have led to the development of a new, targeted therapy, teprotumumab. Teprotumumab is a fully human monoclonal IgG1 antibody that binds with high selectivity and affinity to insulin-like growth factor 1 receptor (IGF-1R). IGF-1R is a ubiquitously expressed transmembrane tyrosine kinase receptor regulating cell growth and proliferation, rather than being a disease-specific stimulatory autoantibody or inflammatory mediator. Nevertheless, IGF-1R is implicated in the pathogenesis of GO on multiple levels [26]. Orbital fibroblasts in GO have been shown to have 3-4x increased expression of IGF-1R compared to control fibroblasts [27]. Other in vitro studies have shown that treating GO orbital fibroblasts with IGF-1 results in increased cell proliferation [28] and can induce hyaluronan synthesis in GO orbital fibroblasts but not in control orbital fibroblasts [29]. Gene expression microarray analysis has also suggested a role for IGF-1R pathway components in active GO [30]. However, it must also be noted, that some of these findings have not been reproduced in other centres [31, 32].

Teprotumumab binds with high selectivity and affinity to IGF-1R, displacing IGF-1 and resulting in internalisation and degradation of the receptor/antibody complex [33]. It reduces cell surface expression levels of IGF-1R and TSHR in fibrocytes from patients with Graves and also reduces TSH-dependent IL-6 and IL-8 expression [34].

Teprotumumab is administered intravenously at an initial dose of 10 mg/ kg thereafter 20 mg/kg every 3 weeks for 21 weeks. The safety and efficacy of teprotumumab in treating patients with active thyroid eye disease has now been studied in two multicentre randomized double-masked placebo-controlled trials: a phase 2 trial (NCT01868997) published in 2017^2^ followed by a phase 3 trial (NCT03298867, OPTIC), the initial results of which were published earlier this year [3]. Both studies have demonstrated a remarkable ability of this drug to reverse diplopia and proptosis [2, 3]. Remarkably, the dramatic improvement in proptosis and diplopia in patients in both trials were similar to those obtained with surgery [2, 26, 35], suggesting that this new treatment could alter current treatment paradigms and be truly disease-modifying.

In the phase 2 trial, 88 patients with recent onset active moderate-to-severe GO were enrolled. The composite primary endpoint was defined as a reduction in CAS of ≥2 points and a reduction of ≥2 mm proptosis in the study eye, without an equal deterioration in the non-study eye. Compared with placebo group, patients treated with tetrotumumab demonstrated a significant reduction in both CAS and proptosis, at 6, 12, 18 and 24 weeks (all *P* < 0.001) [2]. 71.4% of teprotumumab-treated patients (versus 20% of placebo-treated patients) had ≥2 mm reduction in proptosis at week 24 (*p* < 0.001) [2]. The largest improvement was seen in the more severely affected patients [26] with 40% of the teprotumumab-treated patients experiencing a ≥ 4 mm reduction in proptosis compared to none of the patients in the placebo group. Overall, the mean reduction in proptosis (− 3.14 mm) was comparable with results from orbital decompressions (− 3.8 mm) [26].

The findings from the phase 2 trial were recently confirmed with the initial results of the phase 3 trial [3]. Patients were included in this study if they had active moderate-to-severe GO, ocular symptoms that began ≤9 months prior to baseline assessment (making them more likely to be in the active stage) and a CAS score of ≥4 in the more proptotic (study) eye. The primary outcome was defined as a ≥ 2 mm reduction in proptosis at week 24. Patients were randomised to IV teprotumumab (*n* = 41) or placebo (*n* = 42), given according to the same treatment protocol as in the phase 2 study [3].

Improvement in proptosis at week 24 were observed in 83% of the teprotumumab group (vs 10% of the placebo group) (*p* < 0.001) [3]. The mean reduction in proptosis was − 2.82 mm in the teprotumumab group compared to − 0.54 mm in the placebo group, *P* < 0.001^3^ – notably not quite as pronounced a reduction in proptosis as in the phase 2 trial, but still clinically significant. Not only was response to treatment rapid, patients showed continued response at week 28 (7 weeks after the last treatment), without regression of effect [3].

The impact on proptosis has led the FDA to designate teprotumumab with “breakthrough” status. The importance of clinical and subjective improvements in diplopia and proptosis, which previously have required surgical intervention for correction and present significant management challenges, cannot be overstated. However, it must be kept in mind that the sample sizes were relatively small in these trials, and despite the remarkable results achieved in both studies, it remains to be seen whether use of teprotutumab will actually be associated with reduced requirement for rehabilitative strabismus and orbital decompression surgery in real-world settings. Neither trial has yet looked at the impact on rehabilitative surgery in these patients, although reduction in proptosis is considered a good surrogate endpoint for this. In both studies, initial post-treatment assessment was carried out at 24 weeks, just 3 weeks after the final dose of teprotumumab was administered. A longer observation period is required, far beyond the 24 and 28-week outcomes reported in the two trials, will be required to confirm potential benefits on requirements for surgery. In addition, a longer follow-up period would allow investigators to assess for any possible reactivation of inflammatory disease. Further results from the OPTIC trial are expected later this year that may address this [36]. Interestingly, despite the significant reduction in proptosis seen in the teprotumumab group, there was only a modest increase in quality of life in this group. Moreover, there was no statistically significant increase in the appearance-related subscale of quality of life compared to the placebo group, surprising given that one might expect proptosis to be closely linked appearance-related quality of life [37].

A further consideration is whether teprotumumab is as effective in patients with milder forms of GO. The phase 3 trial included only patients with CAS of ≥4, but it is known that a low CAS score does not necessarily exclude inflammatory activity in thyroid eye disease, particularly in cases involving myopathy with diplopia [38]. Whether teprotumumab has a similar effect on patients with active disease but lower CAS scores requires further evaluation. Additionally, most of the participants in the two studies were Caucasian. It remains to be seen whether similar therapeutic effects can be achieved in other ethnic groups.

## A first or second line agent?

While the results of the two teprotumumab trials have shown clinical effects that appear to exceed the effects of current first line treatments, further studies directly comparing teprotumumab with current first line treatments, namely steroids as opposed to placebo, would help to confirm its superiority over these agents. If teprotumumab is indeed more efficacious than glucocorticoids at disease modification, the question that follows is: should teprotumumab replace current first-line agents, or is its role akin to a second-line ‘rescue’ treatment when first line treatments have been unsuccessful at preventing proptosis and/or diplopia? Given the cost of teprotumumab - at $14,900 USD per vial (administered every 3 weeks for a 21 week period), studies directly comparing teprotumumab to current first-line agents would certainly help to inform clinicians of the cost-effectiveness of teprotumumab and its optimal position in the treatment pathway. There is certainly a strong argument for using Teprotumumab as a first line agent in patients with progressive proptosis, given the unique impact on proptosis reversal.

Another consideration for the use of teprotumumab over traditional treatments is the side effect profile. In the phase 2 study, hyperglycemia was the only adverse event clearly identified by the investigators as being related to teprotumumab [2]. In most cases it was mild, and induced hyperglycaemia in patients with diabetes, was readily controlled by adjustments in diabetic medications [2]. Given the large side effect profile of steroids, which includes hyperglycaemia, teprotumumab may be advantageous in this respect. It should be noted that teprotumumab has potential terotogenic effects and its use should be avoided in pregnant women.

## Conclusion

The results from these two large trials have shown that teprotumumab could have remarkable effects on multiple clinical outcomes in GO. It may herald a new era in the treatment of thyroid eye disease and we are cautiously optimistic that teprotumumab may offer an alternative to surgery and its associated complications. Additional studies, including an extension of the phase III OPTIC trial, OPTIC-X (NCT03461211), are currently underway [36] and will continue to shape the treatment of GO and define the role of teprotumumab alongside established treatments.

## Data Availability

Not applicable.
